# Glycemia, Beta-Cell Function and Sensitivity to Insulin in Mildly to Critically Ill Covid-19 Patients

**DOI:** 10.3390/medicina57010068

**Published:** 2021-01-14

**Authors:** Ioannis Ilias, Aristidis Diamantopoulos, Maria Pratikaki, Efthymia Botoula, Edison Jahaj, Nikolaos Athanasiou, Stamatios Tsipilis, Alexandros Zacharis, Alice G. Vassiliou, Dimitra A. Vassiliadi, Anastasia Kotanidou, Stylianos Tsagarakis, Ioanna Dimopoulou

**Affiliations:** 1 Department of Endocrinology, Diabetes and Metabolism, Elena Venizelou Hospital, GR-11521 Athens, Greece; 2 Department of Endocrinology, Diabetes and Metabolism, Evangelismos Hospital, GR-10676 Athens, Greece; aris_dimad@yahoo.gr (A.D.); bclf2008@yahoo.gr (E.B.); dimitra.vas@googlemail.com (D.A.V.); stsagara@otenet.gr (S.T.); 3 Department of Microbiology, Evangelismos Hospital, GR-10676 Athens, Greece; mpratikaki@yahoo.com; 4 First Department of Critical Care Medicine & Pulmonary Services, Medical School of National & Kapodistrian University of Athens, Evangelismos Hospital, GR-10676 Athens, Greece; edison.jahaj@gmail.com (E.J.); nikolaosathanasiou14@gmail.com (N.A.); alexandroszacharis5@gmail.com (A.Z.); alvass75@gmail.com (A.G.V.); akotanid@med.uoa.gr (A.K.); idimo@otenet.gr (I.D.); 5 Department of Pulmonary Medicine, Evangelismos Hospital, GR-10676 Athens, Greece; stamostsipil@gmail.com

**Keywords:** blood glucose, pandemics, severe acute respiratory syndrome coronavirus 2, humans, hyperglycemia, hospitalization

## Abstract

*Background and objectives:* Critically and non-critically ill patients with SARS-CoV-2 infection (Covid-19) may present with higher-than-expected glycemia, even in the absence of diabetes. With this study we aimed to assess glucose, glycemic gap (GlyG) and insulin secretion/sensitivity measures in patients with Covid-19. *Materials and Methods:* We studied, upon admission, 157 patients with Covid-19 (84: in wards and 73: in intensive care units; ICU); 135 had no history of diabetes. We measured blood glucose upon admission as well as glycated hemoglobin (A1c), plasma insulin and C-peptide. We calculated the GlyG and the Homeostasis Model Assessment 2 (HOMA2) estimates of steady state beta cell function (HOMA2%B) and insulin sensitivity (HOMA2%S). Statistical assessment was done with analysis or the Kruskal-Wallis test. *Results:* Compared to patients in the wards without diabetes, patients with diabetes in the wards, as well as patients in the ICU (without or with diabetes) had higher admission glycemia. The GlyG was significantly higher in patients without diabetes in the ICU compared to patients without diabetes in the wards, while HOMA2%B based on glucose and insulin was significantly higher in the ICU patients compared to patients in the wards. Of all the parameters, HOMA2%S based on C-peptide/glucose was higher in survivors (*n* = 133). *Conclusions:* In our series of patients with Covid-19, a substantial number of patients with and without diabetes had admission hyperglycemia and those who were critically ill may have had compromised insulin secretion and lowered sensitivity to insulin. These findings lend credence to reports of association between Covid-19 and hyperglycemia/secondary diabetes.

## 1. Introduction

Infection with coronaviridae may have repercussions in insulin secretion and glycemia [1]. Critically ill and non-critically ill patients with SARS-CoV-2 infection (Covid-19) may present with higher-than-expected glycemia, even in the absence of diabetes [1,2,3,4,5,6,7]. Critically ill patients may show stress hyperglycemia [8]. In patients with diabetes and serious respiratory diseases, the glycemic gap (GlyG; defined by the admission blood glucose measurement minus the average glycemia as calculated from levels of glycated hemoglobin [A1c]) has been associated with prognosis [9,10,11,12]. With this study we aimed to assess glucose, GlyG and insulin secretion/sensitivity measures in patients with Covid-19.

## 2. Materials and Methods

We included in the study patients with polymerase chain reaction (PCR)-verified Covid-19 who were admitted in the wards or the intensive care units (ICU) of the largest Covid-19 referral hospital in Athens, Greece from April 2020 to October 2020. We did not include patients who were initially admitted to the wards and were transferred to the ICU. We measured blood glucose upon admission (presumed to be a fasting measurement, as the patients had not eaten for at least 6–8 h before this event) as well as their A1c upon admission (with the Roche/Hitachi Cobas c 501 analyzer, Rotkreuz ZG, Switzerland). We also measured at the same time plasma insulin and C-peptide (with Immulite 2000 chemiluminescence assays and analyzer, Siemens Healthcare Diagnostics, Inc., Tarrytown, NY, USA). We calculated the GlyG (as follows: GlyG = blood glucose upon admission (in mg/dL) − ([28.7 × HbA1c (in %)] − 46.7)) and the Homeostasis Model Assessment 2 (HOMA2) estimates of steady state beta cell function (HOMA2%B) and insulin sensitivity (HOMA2%S) [13], as percentages of a normal reference population (available from the Diabetes Trials Unit, University of Oxford, Radcliffe Department of Medicine, Oxford, UK, https://www.dtu.ox.ac.uk/homacalculator/). We noted any administration of dexamethasone before blood sampling (if so it was done usually within minutes of the blood sampling and never exceeding an hour) as well as prior medical history or diagnosis/therapy of any form of diabetes (by searching physicians’ documentation and electronic records from the Hellenic National Health service and querying the patients’ next of kin) and the patients’ disease outcome. Analysis of parameters was done with analysis of variance (ANOVA) or the Kruskal-Wallis test (according to the normality/non-normality of their distribution per a Kolmogorov-Smirnov test); statistical significance was set at *p* = 0.05. Power calculations were done post-hoc. Analysis was done with R (R Core Team (2020). R: A language and environment for statistical computing. R Foundation for Statistical Computing, Vienna, Austria. ISBN 3-900051-07-0, URL http://www.R-project.org/). The Scientific Council/Ethics Board of the Evangelismos Hospital (No 170/24 April 2020) gave permission for this study and informed consent for the inclusion of the patients’ anonymized data for study was given by the patients or their next of kin.

## 3. Results

From an initial group of 197 patients (107 ward/90 ICU), data for analysis were available for 157 who were retained (mean age ± standard deviation (SD) = 60.2 ± 15.3 years; 84 in the Covid-19 wards and 73 in the ICU). Demographics and results of the laboratory assessment can be found in Table 1 and Table 2. Most patients had no past medical history of diabetes (*n* = 135). Patients with diabetes were treated with metformin (MTF) and/or inhibitors of dipeptidyl peptidase 4 (DPP4i) and/or sodium-glucose cotransporter-2 inhibitors (SGLT2i); no treatment had been given on the day of admission. Patients in the wards were younger while mortality was much higher in the ICU.

**Figure 1:**
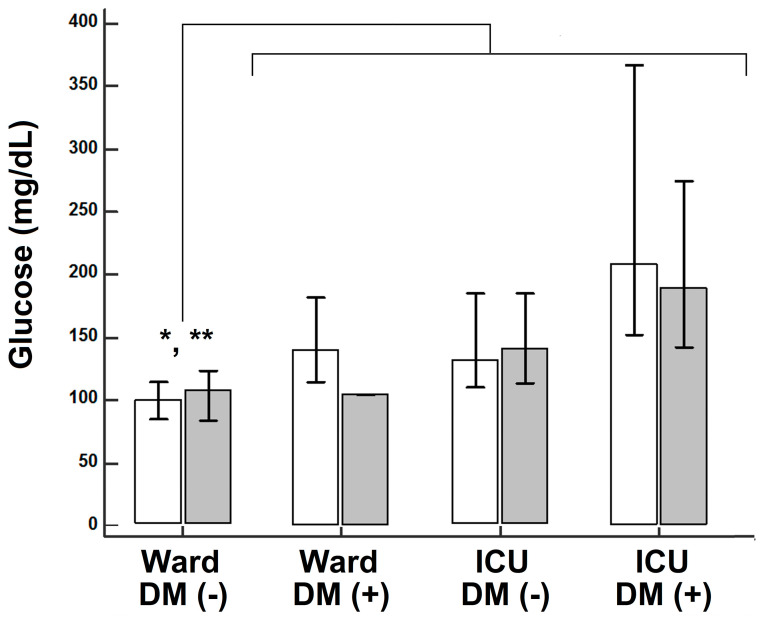
Admission glucose level in patients that did not receive dexamethasone (unshaded) or that received dexamethasone (shaded); * *p* = 0.0077 (no dexamethasone) and ** *p* = 0.00003 (dexamethasone), respectively, Kruskal-Wallis.

Compared to patients in the wards without diabetes, patients with diabetes in the wards, as well as patients in the ICU (without or with diabetes) had higher admission glycemia. Vis-à-vis dexamethasone administration, significant differences were noted only regarding admission glucose (Figure 1).

Nevertheless, either among patients that received dexamethasone or those that did not receive this treatment, patients in the wards with no history of diabetes had significantly lower glucose compared to patients in the ICU and/or positive history of diabetes (Figure 1). There were no other significant differences vis-à-vis the effect of dexamethasone in A1c, GlyG or HOMA indexes (data not shown for brevity). Differences in A1c among the different groups did not reach statistical significance. The GlyG was significantly higher in patients without diabetes in the ICU compared to patients without diabetes in the wards. While insulin, C-peptide and insulin sensitivity indexes showed no significant differences among the study groups, HOMA2%B based on glucose and insulin (but not based on glucose and C-peptide) was significantly higher in the ICU patients compared to patients in the wards. Of all the parameters, HOMA2%S based on C-peptide/glucose was higher (and closer to the normal value of 100%) in survivors (median: 48.8%, first quartile: 32.0%, third quartile: 84.2%) compared to non-survivors (median: 14.0%, first quartile: 12.9%, third quartile: 17.5%; *p* = 0.008, regardless of admission to the ICU/ward or of history of diabetes). The post-hoc power with α = 0.05 was 0.88, 0.44, 0.38 and 0.55 for glycemia, GlyG, HOMA2%B (based on glucose and insulin) and HOMA2%S (based on glucose and C-peptide), respectively.

## 4. Discussion

In our study, Covid-19 patients without diabetes had a relatively higher increase in glycemia, regardless of infection severity, compared to Covid-19 patients with diabetes. Critically ill patients with Covid-19 showed findings consistent with a degree of beta cell compromise; higher insulin sensitivity was associated with survival.

A tentative association between Covid-19 and hyperglycemia has been suggested [1,2,3,4,5,14]; the profound inflammatory activation occurring with Covid-19 has been proposed to be associated with decreased insulin secretion and increased insulin resistance [14,15]. In a previous publication, in critically ill Covid-19 patients without diabetes, we noted that a substantial number of them had hyperglycemia [4], a finding in accordance with studies in China and the United States [2,3,5,16]. Admission hyperglycemia has been associated with Covid-19 chest imaging findings [3].

This is possibly the first-to date-study on GlyG, HOMA2%B and HOMA2%S in Covid-19 patients. There are caveats in our study: on average, one of four critically ill patients with no history of diabetes has stress hyperglycemia (the tentative implicated mechanisms include the release of counter-regulatory hormones, the alteration of insulin receptor signaling caused by inflammation and the inhibition of pancreatic beta-cell function and of endogenous insulin secretion) [8,17]. Thus, this stress hyperglycemia may obscure the glycemic profile of Covid-19 patients. In our patients no effect of dexamethasone administration was noted on the examined parameters [18]; this could be explained by the short time interval between the injection and blood sampling. An effect of antidiabetic medications on beta cell function and sensitivity to insulin was present but we cannot ascertain to what degree; from the literature the effect of MTF and SGLT2i on indices of beta cell function has been reported to be in the magnitude of 10–15% (no appreciable effect has been reported for indices of sensitivity to insulin) [19,20,21]. Furthermore, sample size could be considered to be low, the number of patients with known diabetes was low at 14% of our total sample (interestingly this is closer to the actual prevalence of the disease in the general population) and there was no Covid-19-negative control group for proper comparisons (at the time the data were gathered, we did not seek a non-Covid-19 control group, choosing to hone on the differences between patients with mild and severe Covid-19 rather than between patients with and without Covid-19). The study of hyperglycemia in non-diabetic Covid-19 patients presents a field of ongoing research, particularly with the CoviDIAB Project, an international registry of Covid-19 patients with no history of diabetes that present with hyperglycemia [22].

## 5. Conclusions

In our series of patients with Covid-19, a substantial number of patients with and without diabetes had admission hyperglycemia and those who were critically ill may have had compromised insulin secretion and lowered sensitivity to insulin. These findings lend credence to reports of association between Covid-19 and hyperglycemia/secondary diabetes. The latter may also complicate Covid-19 with superinfection.

## Figures and Tables

**Table 1:** Patients demographics, admission data, outcome; data are given as means ± SD or counts.

Group	Age (Years)	Gender (Men/Women)	BMI	APACHE II	SOFA	DEX (n)	Smoking	Comorbidities	Death
1. Ward No Hx of diabetes (*n* = 75)	56.3 ± 17.4 *	50/25	25.7 ± 1.8	N.A.	N.A.	9	15	Single: 23 More than one: 9	2
2. Ward Hx of diabetes (*n* = 10)	59.3 ± 11.9	7/3	27.8 ± 5.3	N.A.	N.A.	1	2	Single: 6 More than one: 4	0
3. ICU No Hx of diabetes (*n* = 60)	62.9 ± 11.9	44/16	26.1 ± 2.4	13.7 ± 4.7	7.0 ± 2.8	36	17	Single: 22 More than one: 12	17
4. ICU Hx of diabetes (*n* = 12)	71.8 ± 10.8	11/1	27.1 ± 1.9	16.2 ± 6.1	7.4 ± 2.8	8	4	Single: 5 More than one: 4	5

Hx: positive medical history, ΒΜΙ: body mass index, APACHE II: Acute Physiology and Chronic Health Evaluation II score, SOFA: Sequential Organ Failure Assessment Score, N.A.: not applicable, DEX: dexamethasone administration, Comorbidities: Asthma, Chronic Obstructive Pulmonary Disease, Hypertension, Coronary Artery Disease. * *p* = 0.003 (3,4) vs. (1).

**Table 2:** Patients’ laboratory evaluation upon admission; data are given as means ± SD or median (1st/3rd quartile).

Group	Glucose (mg/dL)	A1c (%)	GlyG (mg/dL)	Insulin (μU/mL)	C-Peptide (ng/mL)	HOMA2%Bins (%)	HOMA2%Bc-pept (%)	HOMA2%Sins (%)	HOMA2%Sc-pept (%)
1. Ward No Hx of diabetes (*n* = 75)	101 (84/116) *	5.8 (5.3/6.2)	−14.1 (−38.0/+0.4)	9.4 (5.9/23.8)	2.5 (1.6/4.6)	105.2 ± 63.2	128.4 ± 73.7	87.9 (47.6/122.4)	54.0 (35.1/86.2)
2. Ward Hx of diabetes (*n* = 10)	136 (113/171)	7.4 (6.1/11.0)	-5.6 (−29.9/+5.4)	16.6 (14.9/18.4)	3.7 (2.8/4.7)	89.0 ± 84.5	100.4 ± 116.5	41.9 (39.3/46.9)	25.5 (19.5/29.8)
3. ICU No Hx of diabetes (*n* = 60)	138 (110/184)	5.8 (5.4/7.1)	+5.7 ** (−10.1/+22.0)	8.4 (2.8/16.8)	2.9 (1.5/5.9)	76.9 ± 63.2+	109.9 ± 80.6	64.4 (37.8/99.8)	40.9 (16.8/78.3)
4. ICU Hx of diabetes (*n* = 12)	192 (149/275)	6.1 (5.4/6.5)	+30.2 (+5.5/+74.5)	16.7 (13.6/19.8)	3.4 (1.8/4.4)	44.0 ± 19.0+	59.6 ± 65.3	37.0 (32.8/50.3)	27.0 (24.1/124.1)

Hx: positive medical history, A1c: glycated hemoglobin A1c, GlyG: glycemic gap, HOMA2%Bins: Homeostasis Model Assessment HOMA2 estimate of steady state beta cell function based on glucose and insulin measurements as a % of normal, HOMA2%Bc-pept: HOMA2 estimate of steady state beta cell function based on glucose and C-peptide measurements as a % of normal, HOMA2%Sins: HOMA2 estimate of insulin sensitivity based on glucose and insulin measurements as a % of normal, HOMA2%Sc-pept: HOMA2 estimate of insulin sensitivity based on glucose and C-peptide measurements as a % of normal; * *p* = 0.001 (2,3,4) vs (1), ** *p* = 0.031 (3) vs. (1), +*p* = 0.016 (3&4) vs (1&2).

## Data Availability

Data are available from the corresponding author upon request.
